# Locally Produced BDNF Promotes Sclerotic Change in Alveolar Bone after Nerve Injury

**DOI:** 10.1371/journal.pone.0169201

**Published:** 2017-01-10

**Authors:** Hiroko Ida-Yonemochi, Yurie Yamada, Hiroyuki Yoshikawa, Kenji Seo

**Affiliations:** 1 Division of Anatomy and Cell Biology of the Hard Tissue, Niigata University Graduate School of Medical and Dental Sciences, Gakkocho-dori, Chuo-ku, Niigata, Japan; 2 Division of Dental Anesthesiology, Niigata University Graduate School of Medical and Dental Sciences, Gakkocho-dori, Chuo-ku, Niigata, Japan; Second University of Naples, ITALY

## Abstract

Brain-derived neurotrophic factor (BDNF), which is released due to nerve injury, is known to promote the natural healing of injured nerves. It is often observed that damage of mandibular canal induces local sclerotic changes in alveolar bone. We reported that peripheral nerve injury promotes the local production of BDNF; therefore, it was possible to hypothesize that peripheral nerve injury affects sclerotic changes in the alveolar bone. This study aimed to evaluate the effect of BDNF on osteogenesis using *in vitro* osteoblast-lineage cell culture and an *in vivo* rat osteotomy model. MC3T3-E1 cells were cultured with BDNF and were examined for cell proliferative activity, chemotaxis and mRNA expression levels of osteoblast differentiation markers. For *in vivo* study, inferior alveolar nerve (IAN) injury experiments and mandibular cortical osteotomy were performed using a rat model. In the osteotomy model, exogenous BDNF was applied to bone surfaces after corticotomy of the mandible, and we morphologically analyzed the new bone formation. As a result, mRNA expression of osteoblast differentiation marker, osteocalcin, was significantly increased by BDNF, although cell proliferation and migration were not affected. In the *in vivo* study, osteopontin-positive new bone formation was significantly accelerated in the BDNF-grafted groups, and active bone remodeling, involving trkB-positive osteoblasts and osteocytes, continued after 28 days. In conclusion, BDNF stimulated the differentiation of MC3T3-E1 cells and it promoted new bone formation and maturation. These results suggested that local BDNF produced by peripheral nerve injury contributes to accelerating sclerotic changes in the alveolar bone.

## Introduction

**Fig 1:**
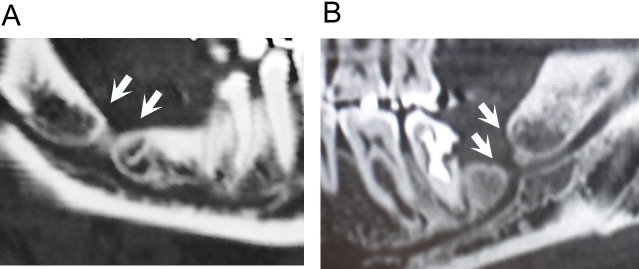
Sclerotic changes in alveolar bone after IAN injury. CT, X-ray and photographic images from patients of a post-extraction tooth cavity. (A) A thirty-five-year-old woman reported dysesthesia for a long period after the tooth extraction. Sclerotic changes were observed in the periphery of the alveolar bone (arrows). (B) A 46-year-old woman experienced dysesthesia, which increased in relation to the deformity of the mandibular canal. In both of these patients, the masses of traumatic neuroma located in the nerve lesion were detected later by surgical observation.

Dental treatments, such as third molar tooth extraction, oral surgeries, local anesthesia injection and implant placement, sometimes cause inferior alveolar nerve (IAN) injury. Clinically, we can observe a delay in natural healing of the tooth-extracted cavity after IAN injury and radiographic findings regarding some delays in bone regeneration with neurological symptoms such as sensory impairment and dysesthesia have sometimes associated with sclerotic changes in alveolar bone and/or deformity of the mandibular canal (Fig 1) [1,2]. We have already reported that peripheral nerve injury results in the release of brain-derived neurotrophic factor (BDNF) locally, and this factor can also promote traumatic neuroma formation [3]. However, it remains unclear whether these clinical, radiographic findings have been derived from peripheral nerve injury.

BDNF is a family of neurotrophic factors, and it serves a function in the survival and differentiation of central and peripheral neurons [4]. BDNF is synthesized by neuronal cells, osteoblasts, endothelial cells, monocytes and fibroblasts [5–8], and it acts by binding to high-affinity tropomyosin-related kinase B receptor (trkB) or p75-NTR receptor [4,9]. It was reported that BDNF appeared in nerve injury lesions in the mandible, and fibroblasts yielded it due to the initiation of nerve injury [3]. Regarding the effect of BDNF on osteogenesis, BDNF is known to promote bone formation during fracture healing in humans [10,11]. However, there have been no reports referring to osteogenic effects of BDNF in association with nerve tissue injury or analyzing the effects of local BDNF using *in vivo* experimental models of IAN injury and osteotomy.

Regarding the relationship between the development of mandibular bone and IAN, the formation of a mandibular canal is known to follow IAN development [12,13]. The mandibular canal, which covers IAN tissue, has a compact bony wall even within the cancellous bone [12,14], and it is suggesting that IAN might affect compact bone formation. Surgical approaches in the vicinity of the mandibular canal sometimes invade the IAN, resulting in peripheral neuropathy. Long-term dysesthesia in this lesion suggests traumatic neuroma formation, as well as a delay in wound healing in the postoperative alveolar bone cavity. Lesions with delayed healing are formed by coverage of the bony wall, which is connected to the mandibular canal and is also connected to post-extraction tooth cavity. This post-tooth extraction space exhibits an inflated shape, suggesting the existence of some tissue in it, and frequently observed the existence of neuromas in it [15–17]. In contrast, we demonstrated that the local administration of anti-BDNF antibody inhibited neuroma formation in rats [9] and introduce of post injured decrease in the withdraw threshold suggesting allodynia [18]. Therefore, these findings suggested that local BDNF produced by nerve injury could be related to two phenomena: neuroma formation and a delay in bony refilling of post tooth-extraction alveolar space. Furthermore, radiological findings of deformities of mandibular canals after nerve injury exhibited structural connections to unhealed spaces with thickened bony walls (Fig 1). These findings indicated that nerve injury induced bone sclerosis in the surrounding bone trabeculae. There have been several reports describing the involvement of nerve tissue in bone formation, and neuron-derived factors, such as nerve growth factor (NGF) and semaphorin 3A, are known to regulate bone remodeling directly or indirectly by modulating sensory nerve development [19]. Therefore, we believe that it is important to consider the relationship between bone formation and nerve regeneration.

This study aimed to prove the biological effect of BDNF on bone remodeling using *in vitro* cell culture and *in vivo* cortical osteotomy experiments in the rat mandible, as well as to elucidate the causes of sclerotic changes in the surrounding bone after IAN injury.

## Materials and Methods

### Cells and cell culture

Murine MC3T3-E1 pre-osteoblast cell lines were utilized for *in vitro* studies [20]. Cells were grown in α-minimum essential medium (MEM) (Gibco BRL, Grand Island, NY, USA) with 10% fetal bovine serum and 1% penicillin–streptomycin (Gibco BRL) in 5% CO_2_ at 37°C. The culture medium was changed every 2 days.

### Cell proliferation assay

To analyze the effects of BDNF on cell proliferation, MC3T3-E1 cells were plated in 6-well plates at density of 5 x 10^3^/well and were incubated in the presence or absence of BDNF (80 ng/ml, Miltenyi Biotec Inc., CA, USA). Firstly, we tried *in vitro* experiments using 50, 80 and 100 ng/ml of BDNF, and we determined the most effective concentration of BDNF as 80 ng/ml (data not shown). At 1, 3, 5, and 7 days after adding BDNF, the cells were collected by 0.25% trypsin, and the total number of the cells was counted using a hemocytometer. We repeated the same assays more than 3 times for each condition.

### *In vitro* scratch assay

An artificial wound was generated on confluent monolayers of MC3T3-E1 cells in 12-well culture plates with a scratch stick (Asahi Glass Co., Ltd., Shizuoka, Japan). One day and three days after plating, the cells were fixed with 4% paraformaldehyde in 0.1 M phosphate buffer (pH 7.4) and were stained with toluidine blue. The rate of migration was measured by quantifying the distances that the cells moved from the edge of the scratch toward the center of the scratch [21]. We measured five places per well, and the average values of the obtained measured data were statistically analyzed as described below.

### Reverse transcriptase-polymerase chain reaction (RT-PCR)

MC3T3-E1 cells were grown in the osteogenic medium containing 10 mM β-glycerophosphate (Wako Pure Chemical Industries, Ltd., Osaka, Japan), 50 μg/ml ascorbic acid (Wako), and 100 nM dexamethasone (Sigma-Aldrich, St. Louis, MO, USA), with or without BDNF (80 ng/ml). After 5 days, total RNA was isolated from MC3T3-E1 cells using the Trizol system (Invitrogen Corporation, Carlsbad, CA, USA). cDNA was synthesized from the RNA with the SuperScript First-Strand Synthesis System (Invitrogen). The sequences of the PCR primer pairs for *β-actin*, *alkaline phosphatase (ALP)*, *osteopontin*, *osteocalcin*, *bcl-2*, *caspase 3* and *cyclin D1* are listed in S1 Table. The thermocycling protocol during 30 amplification cycles was as follows: denaturation at 94°C for 1 minute, annealing at 60°C for 1 minute, extension at 72°C for 1 minute. The amplified DNA fragments were separated by electrophoresis on 2% agarose gels.

### Immunocytochemistry

For immunofluorescent staining, MC3T3-E1 cells were plated in 8 well Lab-Tek II chamber slides (Nalge Nunc. International, Roskilde, Denmark) and were incubated with BDNF (80 ng/ml) for 30 min, 2 or 5 days. The cells were fixed with 4% paraformaldehyde in 0.1 M phosphate buffer (pH 7.4) and were immunostained using polyclonal antibodies against trkB (H-181, Santa Cruz, CA, USA), osteopontin (LSL-LB-4225, Cosmo Bio Co., Tokyo, Japan), and anti-phospho-Akt (Ser473; Cell Signaling Technology, Danver, MA, USA). FITC-conjugated anti-rabbit IgG (diluted to 1:500; Vector Laboratories, Burlingame, CA, USA) was used as a secondary antibody. The stained cells were analyzed with a confocal laser scanning microscope (FV300, Olympus, Tokyo, Japan) or a fluorescence microscope (BX51, Olympus, Tokyo, Japan).

### Von kossa staining

MC3T3-E1 cells were grown in the osteogenic medium with or without BDNF (80 ng/ml). After 7 days, cells were fixed with 4% paraformaldehyde in a 0.1 M phosphate buffer (pH 7.4), and treated with a 5% solution of AgNO_3_ for 1 h and with a 5% solution of sodium thiosulfate for 3 min.

### Western blotting

MC3T3-E1 cells were lysed in RIPA buffer (50 mM Tris pH 7.5, 150 mM NaCl, 1 mM EDTA, 1% Triton X-100, and 1% phosphatase inhibitor cocktail [Nacalai Tesque, Kyoto, Japan]). The cell lysates were separated by SDS-polyacrylamide gel electrophoresis and then were electrophoretically transferred to PVDF membranes (Bio-Rad, MA, USA). For immunodetection, the following antibodies were used: anti-β-tubulin (Sigma-Aldrich), anti-Akt (Cell Signaling Technology), anti-phospho-Akt (Ser473; Cell Signaling Technology), anti-ERK1+ERK2 (Abcam Inc., Boston, USA) and anti-phospho-ERK (Tyr 204: Santa Cruz, CA, USA). The Envision+/HRP system (Dako, Glostrup, Denmark) was used and protein bands were visualized by 0.02% 3,3’-diaminobenzidine (Dohjin Laboratories, Kumamoto, Japan). The relative densities of each band against that of β-tubulin on monochrome photographs were determined with Image J software (Image J 1.45, NIH, USA).

### IAN transection experiment of the rat mandible

All the animal experiments were conducted in compliance with the protocol which was reviewed by the Niigata University Intramural Animal Use and Care Committee and approved by the President of Niigata University (Niigata Univ. Res.232-2). Eight and ten-week-old male Sprague-Dawley rats (Charles River Laboratories Japan, Inc., Yokohama, Japan) were housed at 25°C and approximately 40% humidity, with a 12-h light/dark cycle and free access to food and water. In order to demonstrate the expression of BDNF after IAN injury, we performed IAN transection experiments using the 8-week-old rats. In the animals with nerve lesion, the surface of the mandible was removed to expose the IAN, which was transected completely with microscissors. In the sham operated group, IAN was exposed but without transection. This was conducted according to previous reports [3,18].

### Cortical osteotomy of the rat mandible with exogenous BDNF

In order to examine the effect of BDNF on bone remodeling among some neuron-derived factors such as NGF and semaphorin, we performed cortical osteotomy experiment with exogenous BDNF. Twenty-four 10-week-old male Sprague-Dawley rats were used in this study. The animals were divided into two groups: BDNF treatment (n = 12) and vehicle control (n = 12). The rats were deeply anesthetized by inhalation of sevoflurane, and intraperitoneal injections of chloral hydrate (350 mg/kg) were added by the depth of anesthesia by pinching the paws. A small incision was made in the skin of the left cheek, and the surface cortical bone of the mandible was removed. BDNF (5 μg/2 μl) was injected into the cavity made by the corticotomy, using a Hamilton syringe (Hamilton Company, Reno, NV, USA). After the local administration to spaces of the lesion, it was covered by bone wax in order to prevent from the leakage of administered BDNF. This procedure was conducted according to our previous reports [3,18]. And then the incisions were closed with sutures. After 7 days, 14 days and 28 days, all rats were placed under deep anesthesia with sevoflurane and perfused transcardially with physiological saline, followed by 4% paraformaldehyde in 0.1 M phosphate buffer (pH 7.4). The heads were immersed in the same fixative for an additional 12 hours and were scanned for micro–computed tomography (Elescan; Nittetsu Elex, Tokyo, Japan). Buccal cortical bone areas surrounding osteotomy injury within 1800 μm^2^ were measured using graphics software (Adobe Photoshop CS4 for Mac, Adobe Systems Incorporated, San Jose, CA, USA) for all samples, and the average values of the obtained measured data were statistically analyzed as described below. Following decalcification in a 10% ethylenediamine tetraacetic acid disodium salt (EDTA-2Na) solution for 2 weeks at 4°C, serial sections 4 μm in thickness were stained with hematoxylin-eosin (HE) and were processed for azocarmine and antiline blue (Azan) staining or immunohistochemistry using the antibodies described below.

### Immunohistochemistry

Immunohistochemistry was performed using the Envision+/HRP system (Dako) with rabbit anti-ALP [22], rabbit anti-osteopontin (LSL), rabbit anti-trkB (Santa Cruz) and mouse anti-cathepsin K (Daiichi Fine Chemical Co., Ltd, Toyama, Japan) antibodies. Sections were treated with 0.3% hydrogen peroxide in methanol for 30 minutes at room temperature to block endogenous peroxidase activity. After rinsing in PBS, the sections were incubated with 5% skimmed milk in PBS and 0.05% Triton X-100 (T-PBS) for 1 hour at room temperature to block non-specific protein-binding sites. They were then incubated overnight at 4°C with the primary antibodies diluted at 50 μg/ml in T-PBS. For visualization of the reaction products, sections were the treated with 0.02% 3,3’-diaminobenzidine (Dohjin Laboratories) in 0.05 M Tris-HCl buffer (pH 7.4) containing 0.005% hydrogen peroxide, and they were counterstained with hematoxylin. For control experiments, the primary antibodies were replaced with preimmune rabbit or mouse IgG.

### Statistical analysis

Mann-Whitney *U* test was performed using statistical software (StatPlus for Mac, Japan) for all of the experiments. The numbers of cathepsin K-, ALP- and trkB- positive cells on the bone surfaces of the central areas of each specimen (a grid [357 x 238 μm^2^] was selected) were calculated separately. Osteopontin-positive bone areas in the central areas of each specimen (a grid [357 x 238 μm^2^] was selected) were calculated using graphics software (Adobe Photoshop CS4 for Mac). Data were obtained from 72 areas of 24 samples (control: 36 areas, n = 12, BDNF: 36 areas, n = 12) in the osteotomy experiments. All of the data are presented as the means and standard deviations (SDs) of each group. A difference was considered as significant if the p value was less than 0.05 level.

## Results

### BDNF localizes in the injured nerve tissue after IAN injury in rat

**Fig 2:**
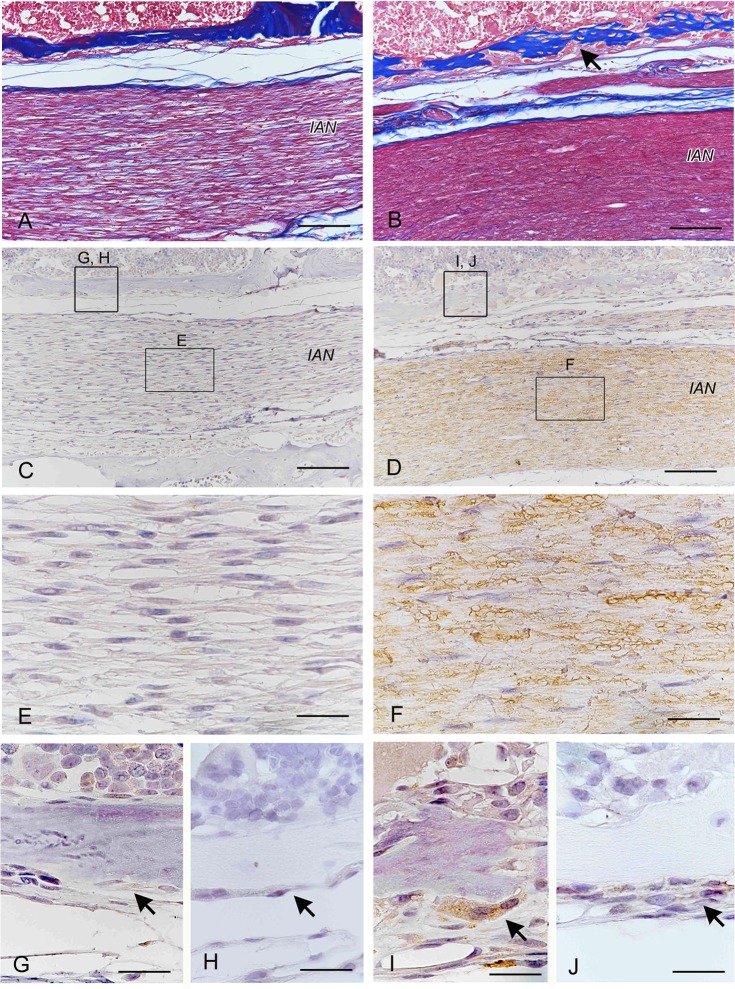
BDNF localizes in the injured IAN. Histological analysis of inferior alveolar nerve (IAN) injury experiments in rats after 14 days. Azan staining (A, B) and immunohistochemical stainings of BDNF (C-G, I) and trkB (H, J). (A, C, E, G, H) Unaffected region of IAN injury. (B, D, F, I, J) Regenerating IAN region after injury. Although unaffected IAN and peripheral bone tissues did not express BDNF (C, E, G), injured IAN (D, F) and active osteoblasts on the surface of newly formed bone (I) were immunopositive for BDNF. The osteoblast lineage cells surrounding newly formed bone were immunopositive for trkB (J). *IAN*; inferior alveolar nerve. Bars, 100 μm (A-F), 20 μm (G-J).

We performed IAN transection experiments to examine the expression of BDNF and involvement of BDNF during bone healing after IAN injury. At 14 days after surgery, the nerve tissue regenerated and new bone formation was observed in the vicinity of the injured nerve lesion (Fig 2B). Although unaffected nerve fibers and peripheral bone tissues did not express BDNF (Fig 2A, 2C, 2E and 2G), injured nerve tissue (Fig 2D and 2F) and active osteoblasts on the surface of newly formed bone (Fig 2I) were strongly immunopositive for BDNF. Although the expression of trkB was not observed in osteoblasts of existing bone wall (Fig 2H), the osteoblast lineage cells surrounding newly formed bone were immunopositive for trkB (Fig 2J).

### BDNF promotes osteoblast differentiation *in vitro*

**Fig 3:**
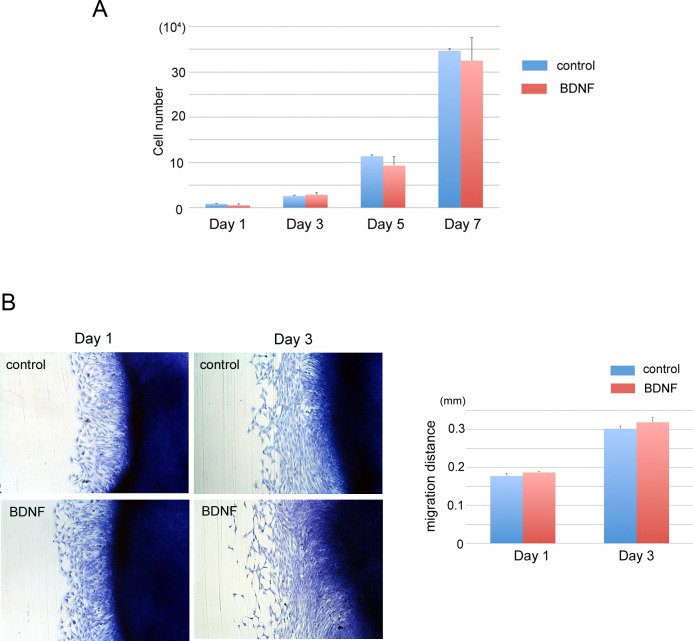
BDNF did not affect cell proliferation and migration in MC3T3-E1 cells. MC3T3-E1 cells were treated with recombinant BDNF. (A) Cell proliferation assay. BDNF did not affect the proliferation of MC3T3-E1 cells. (B) Cell migration assay. BDNF did not promote cell movement according to *in vitro* scratch assay.

**Fig 4:**
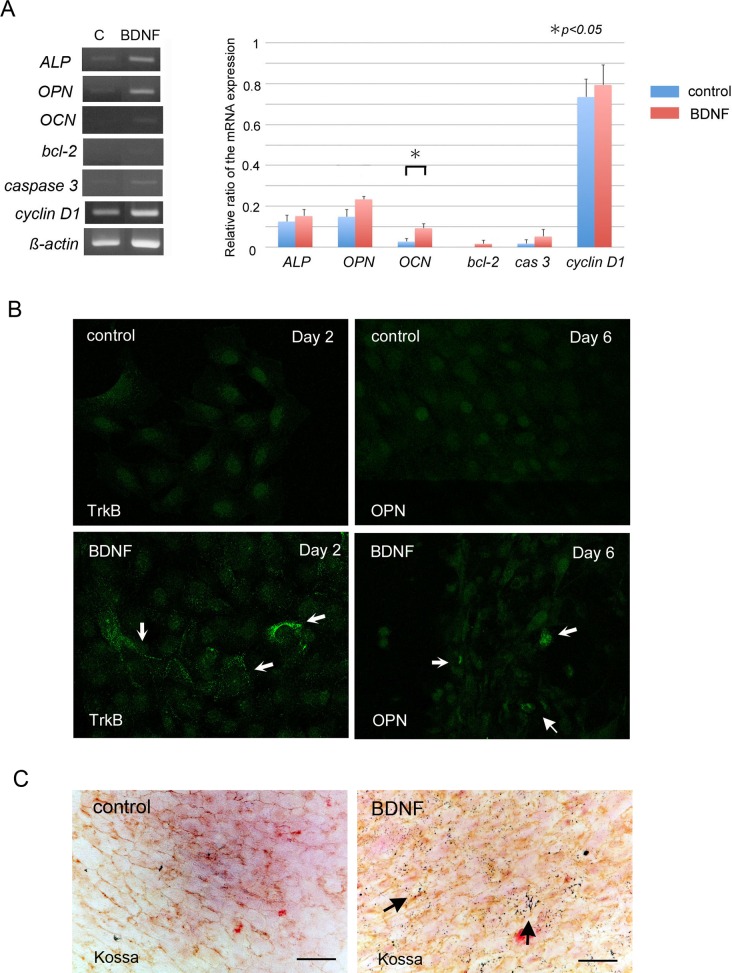
BDNF promotes osteoblastic differentiation in MC3T3-E1 cells. MC3T3-E1 cells were treated with recombinant BDNF. (A) RT-PCR analysis. mRNA expression level of osteocalcin was significantly increased after treatment with BDNF. (B) Immunofluorescent staining of trkB and OPN. BDNF upregulated trkB and OPN immunoreactivity in MC3T3-E1 cells. (C) BDNF enhanced mineralization of MC3T3-E1 cells after 7 days. Calcium deposition was stained with black dots by von Kossa staining (arrows) (C). Bars, 20 μm (C).

MC3T3-E1 cells were used to examine the effects of BDNF on osteoblast proliferation and differentiation *in vitro*. BDNF did not affect proliferation of MC3T3-E1 cells (Fig 3A) and did not promote cell movement by *in vitro* scratch assay (Fig 3B). Next, to determine the effect of BDNF on differentiation toward mature osteoblasts, we performed RT-PCR analysis and immunofluorescent staining using differentiation of molecular markers of osteoblast-lineage cells. The mRNA expressions of osteocalcin in MC3T3-E1 cells were significantly upregulated by BDNF. Cyclin D1, which is associated with cell proliferation, and apoptosis-related markers, such as bcl-2 and caspase 3, were not changed by BDNF (Fig 4A). By immunocytochemical staining, trkB expression was enhanced by BDNF at day 2, and the deposition of osteopontin matrices was observed at day 6 in the BDNF-treated cells (Fig 4B). Von Kossa staining demonstrated an increase in the number of mineralized granules in the presence of BDNF (Fig 4C).

**Fig 5:**
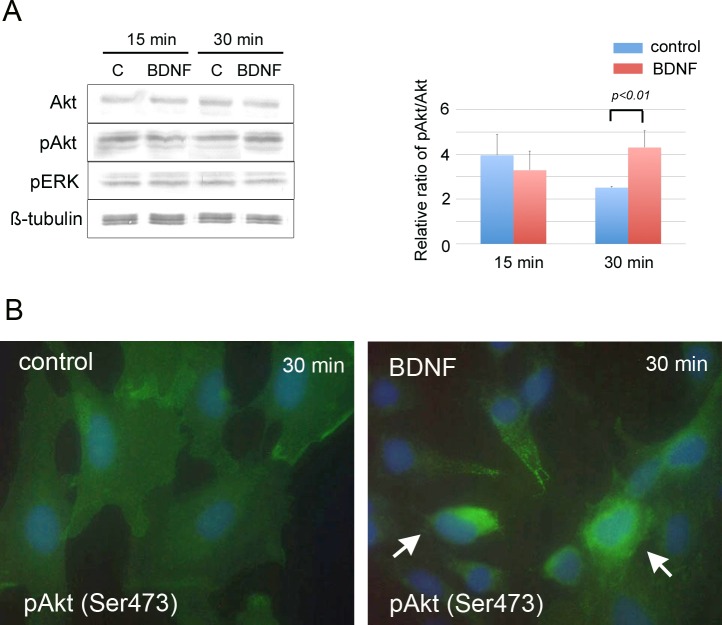
BDNF promotes activation of Akt signals in MC3T3-E1 cells. Western blot analysis (A) and immunocytochemical staining of Akt and pAkt (Ser473) (B) in MC3T3-E1 cells. (A) BDNF significantly increased the active form of Akt (pAkt) in MC3T3-E1 cells at 30 min. On the other hand, the phosphorylation of ERK was not affected by BDNF. (B) BDNF enhanced pAkt immunoreactivity in the cytoplasm. The nucleus is stained with DAPI (blue).

To examine the effects of BDNF on intercellular signaling, MC3T3-E1 cells were treated with BDNF for 15 and 30 minutes. BDNF significantly induced the phosphorylation of Akt at 30 minutes (Fig 5A and 5B). On the other hand, BDNF did not affect the phosphorylation of ERK (Fig 5A).

### BDNF enhances bone remodeling after cortical osteotomy of the mandible

**Fig 6:**
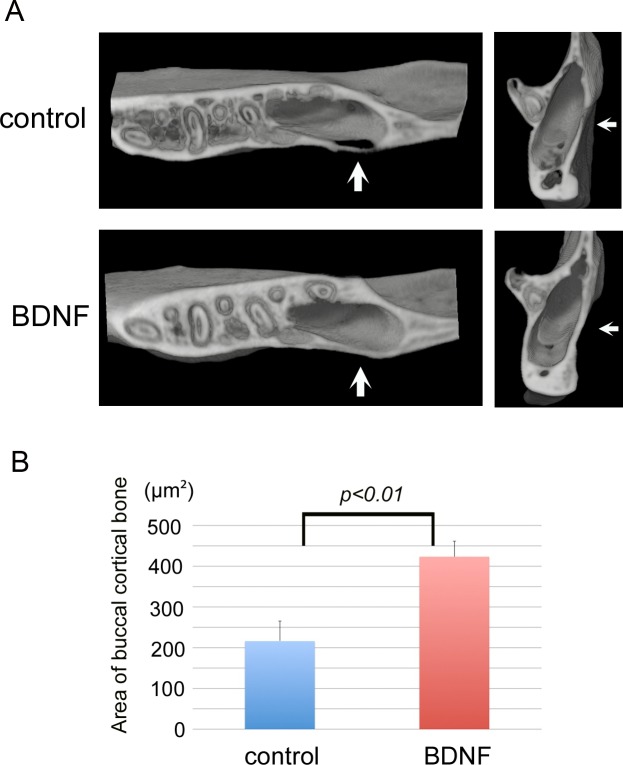
Local BDNF promotes bone formation after osteotomy in the mandible. Micro-computed tomography images in the osteotomy at day 28 in rat. (A) The area of osteotomy was indicated by arrow (A). Twenty-eight days after osteotomy, exogenous BDNF accelerated regeneration of the cortex and induced osteosclerotic changes in the surrounding cortical bones (A). (B) Buccal cortical bone areas around osteotomy injury within 1,800 μm^2^ were measured using graphics software. The bone area was significantly wider in BDNF treated group.

**Fig 7:**
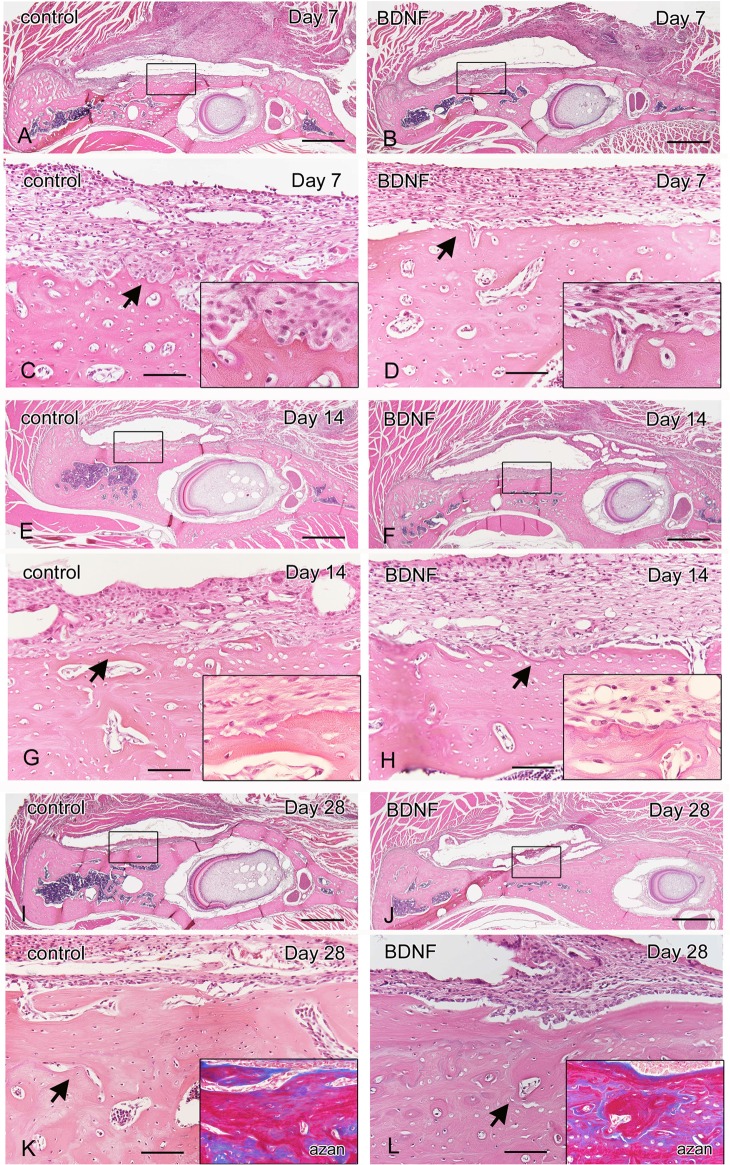
Local BDNF induces osteosclerotic changes to surrounding bones. Hematoxylin and eosin staining (A-L) and azan staining (K, L) on day 7 (A-D), day 14 (E-H) and day 28 (I-L) after osteotomy in rat. (C, D, G, H, K, L) Magnified views of square areas in A, B, E, F, I, J. Histological examination demonstrated that many resorption lacunae were observed on the bone surface at day 7 in the control group (C, arrow and square). In contrast, the cortical bone surface was smooth with few resorption lacunae in BDNF group (D). After day 14, active osteoblasts lined the surface of the BDNF-treated bone (H, arrow and square), although osteoblast differentiation was not prominent in control (G). At day 28, a number of complicated reversal lines were observed in the BDNF-treated bone, and active bone formation occurred not only on the outer surface of the cortical bone but also inside of the bone lacunae and bone marrow cavity as clearly demonstrated by azan staining (L, arrow and square). Bars, 1000 μm (A, B, E, F, I, J), 100 μm (C, D, G, H, K, L).

**Fig 8:**
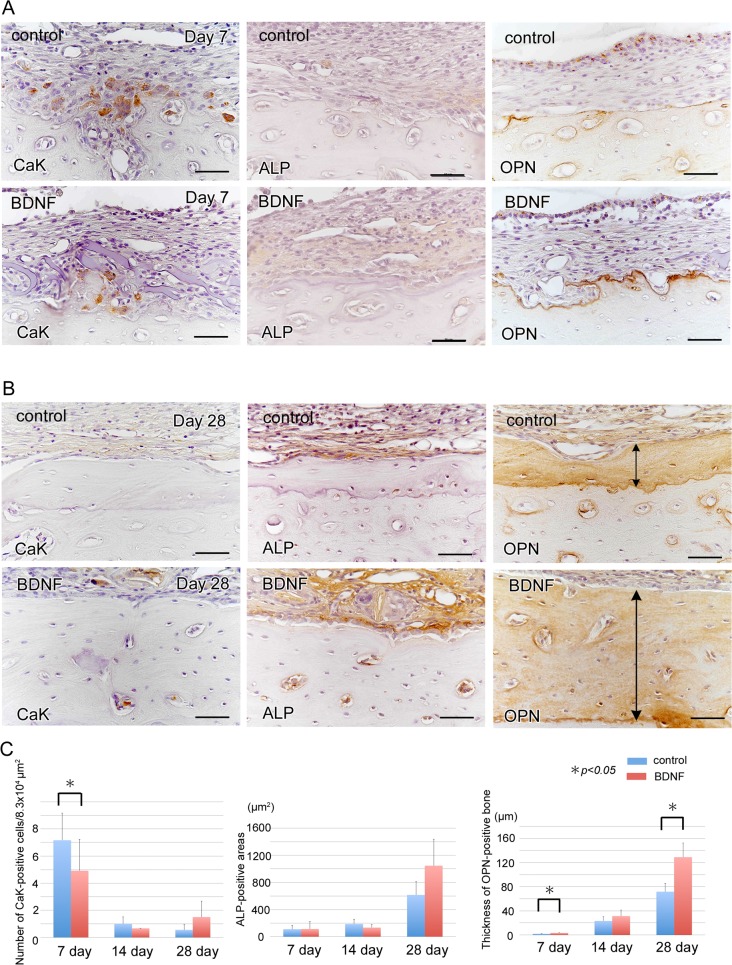
Prolonged active bone remodeling due to local BDNF. Immunohistochemical staining of cathepsin K, ALP and osteopontin at day 7 (A) and day 28 (B) after osteotomy. Many cathepsin K-positive osteoclasts appeared in the control group at day 7. In contrast, osteopontin-positive lines were prominent in the BDNF-treated bone surface (A, C). At day 28, cathepsin K-positive osteoclasts were scattered on the surface of newly formed bone and bone lacunae in the BDNF group. In addition, osteoblast-lineage cells were strongly immunopositive for ALP, and OPN-positive new bone matrix (arrows) was significantly thicker in the BDNF group (B, C). Bars, 50 μm.

**Fig 9:**
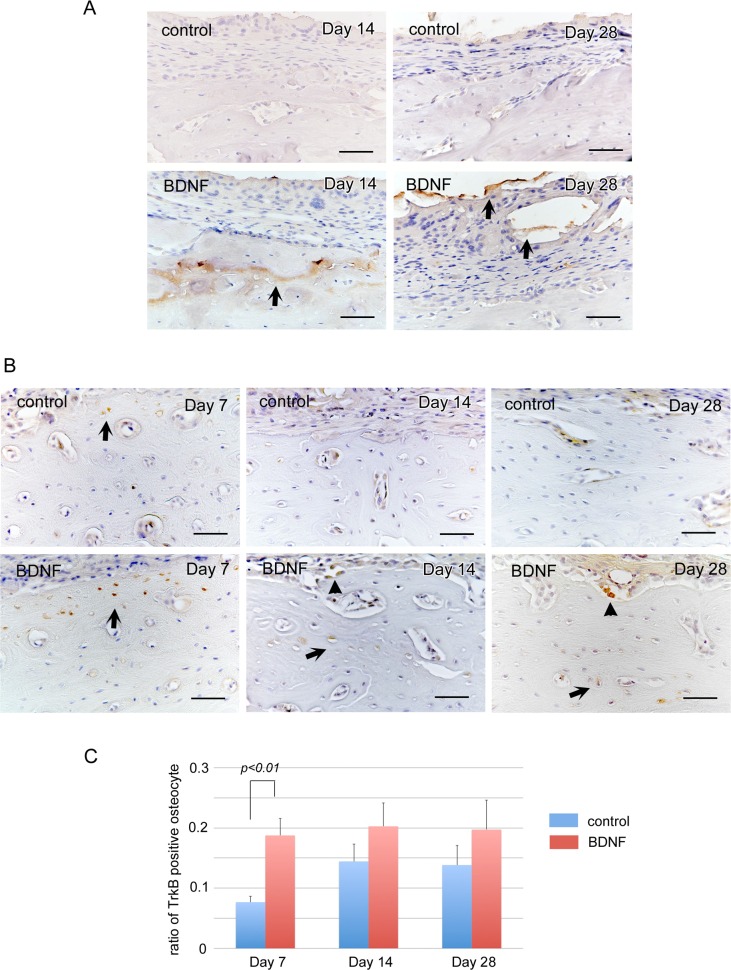
Strong expression of trkB in osteoblasts and osteocytes in response to exogenous BDNF. Immunohistochemical staining of BDNF at day 14 and day 28 (A) and trkB at day 7, day 14 and day 28 (B) after osteotomy. BDNF was deposited within newly formed bone matrices at day 14 and day 28 in the BDNF-treated group (A, arrows). TrkB-positive osteocytes were few in the control group (B). On the other hand, the strongly positive immunoreaction of trkB was observed in the osteoblasts (arrowheads) and osteocytes in bone surface and subsurface area (arrows) at day 7, day 14 and day 28 in the BDNF-treated bones (B). The number of trkB-positive osteocytes in the bone surface area was significantly higher in BDNF group at day 7 (C). Bars, 50 μm.

To examine the osteogenic potential of BDNF *in vivo*, we examined whether or not exogenous BDNF could promote bone repair, using a cortical osteotomy model of the rat mandible. Twenty-eight days after surgery, μCT analysis indicated that exogenous BDNF significantly accelerated the regeneration of the cortex and induced osteosclerosis surrounding the bones as indicated by arrows (Fig 6A and 6B). Histological examination demonstrated that bone defect areas were filled with fibrous tissue in both groups (Fig 7A and 7B), and many resorption lacunae were observed on the bone surface at day 7 in the control group (Fig 7A and 7C). In contrast, the cortical bone surface was smooth without prominent resorption lacunae at day 7 in the BDNF group (Fig 7B and 7D). After day 14, bone resorption by osteoclasts continued as indicated by magnified view (Fig 7E and 7G), and osteoblast differentiation was not prominent in the control group (Fig 7G). On the contrary, active osteoblasts lined on the surface of the BDNF-treated bone (Fig 7F and 7H). At day 28, a number of complicated reversal lines were observed in the BDNF-treated bone, indicating that active bone formation occurred not only on the outer surface of the cortical bone but also inside of the bone lacunae and bone marrow cavity (Fig 7J and 7L) unlike the control bone (Fig 7I and 7K), as clearly demonstrated by azan staining. Next to confirm bone remodeling activity, we performed immunohistochemical staining using the markers for bone resorption and formation. At day 7, many cathepsin K-positive osteoclasts infiltrated in the bone surface in the control group. In contrast, osteopontin-positive lines in the bone surface were prominent in the BDNF-treated group (Fig 8A and 8C). ALP-positive osteoblasts in the bone surface were not obvious in both groups at day 7. At day 28, cathepsin K-positive osteoclasts were scattered near the surface of the newly formed bone in the BDNF group, although there were few in the control group (Fig 8B). In addition, the osteoblast-lineage cells and fibrous tissue covering the bone surface were strongly immunopositive for ALP in the BDNF group. OPN-positive newly bone matrix was significantly thicker in the BDNF group, comparing with the control group (Fig 8B and 8C). Next, to confirm the cells that responded to exogenous BDNF, we checked the expression of BDNF and trkB by immunohistochemistry. In the control group, BDNF was not localized in the injured area at day 14 and day 28 (Fig 9A). In contrast, BDNF was deposited within newly formed bone matrices at 14 days in BDNF-treated bone, and it still retained in the fibrous tissues in the bone surface at 28 days as indicated by arrows (Fig 9A). Regarding trkB expression, trkB-positive cells were few at day 7, day 14 and day 28 in the control group. On the other hand, the strongly positive immunoreaction of trkB was observed in the osteocytes of bone surface at day 7, and lining osteoblasts as well as osteocytes in the subsurface area expressed trkB at day 14 and day 28 in the BDNF-treated group (Fig 9B). The number of trkB-positive osteocytes was significantly higher in the BDNF group at day 7 (Fig 9C).

## Discussion

The present study demonstrated that exogenous BDNF activated the cell differentiation of osteoblast lineage cells *in vitro*, and it promoted new bone formation and enhanced bone remodeling after cortical osteotomy *in vivo*. This report is the first to discuss the cause of sclerotic changes in mandibular bone surrounding IAN lesions after nerve trauma. In our IAN injury model in rats, injured nerve tissue was strongly immunopositive for BDNF and trkB-positive osteoblasts were observed during the healing process of cancellous bones. Therefore, we believe that BDNF, which is secreted locally and transiently due to nerve injury, accelerates bone regeneration surrounding nerve lesions, resulting in sclerotic changes in the alveolar bone after IAN injury. This could be extrapolated from clinical situations.

Regarding the effect of BDNF on bone, several studies have indicated that BDNF has positive effects on bone formation and regeneration in *in vivo* bone fracture models [10,11,23,24]. In our *in vitro* study, mRNA expression of osteoblast differentiation marker, osteocalcin, was significantly increased by BDNF, although cell proliferation and migration were not affected in MC3T3-E1 cells. Similarly, Yada *et al*. reported that NGF stimulated differentiation of MC3T3-E1 cells without affecting cell proliferation [25]. The acceleration of osteoblast differentiation without the proliferation of osteoblast-lineage cells enhanced the peripheral bone healing process over preexisting bone walls in the extracted tooth cavities if peripheral nerve get injured. This can be explained by the fact that the injury induced local release of BDNF, and BDNF spreads to the surfaces of the bone walls. Additionally, in an *in vivo* model, activated osteoblasts that were strongly immunopositive for ALP remained at 28 days after corticotomy in the BDNF group, and thick, OPN-positive new bone matrix deposition was observed in the surface layer of the preexisting bone wall. Simultaneously, cathepsin K-positive osteoclasts continued to infiltrate the bone surface in the BDNF group. Both osteoblasts and osteocytes on the bone surface continued to express trkB strongly at 28 days. These results indicated that osteoblasts, as well as osteocytes, continued to receive exogenous BDNF signals, resulting in the persistence of active bone remodeling associated with narrowing of the bone marrow space and bone lacunae. Osteocytes are known to regulate bone remodeling and adaptation via cell-cell interactions with osteoclasts and osteoblasts in pathophysiological situations [26]. Therefore, continuous trkB expression of osteocytes might be associated with long-term, continuing sclerotic changes in alveolar bone in the vicinity of peripheral nerve injury.

When BDNF binds to trkB, it triggers intracellular survival signaling pathways such as PI3-kinase/Akt signals and MAPK/ERK signals [24,27], whereas binding to p75-NTR induces apoptosis of the cells [11,28]. TrkB is known to be recruited to the cell surface from the cytoplasm by extracellular BDNF stimulation [9], and in our *in vitro* experiment, BDNF enhanced trkB expression on the cell surface and promoted the Akt signaling cascade in MC3T3-E1 cells. Regarding the relationship between Akt signaling and osteoblast differentiation, the PI3K/Akt signaling pathway is known to regulate osteoblast differentiation [29–32]. Therefore, it is speculated that the promotion of osteoblast differentiation by exogenous BDNF occurs through trkB receptor by activating the Akt signaling cascade.

Recently, BDNF has been considered as a potential therapeutic for some neurogenic disorders [33] and periodontal diseases [23]. Jimbo *et al*. applied recombinant BDNF to periodontal furcation bone defects in non-human primates and showed that BDNF promoted alveolar bone regeneration [23]. These reports supported the possibility of local BDNF control for bone regeneration and/or normal healing of bone defects caused by tooth extraction. In contrast, we already reported that BDNF also promoted peripheral nerve growth and could induce traumatic neuroma formation, and trkB mRNA was expressed by fibroblasts within the connective tissue during the regeneration period [3]. Deficiencies in the BDNF-trkB signaling pathway render neuroma formation difficult, and even the peripheral nerve is completely transected, suggesting that this signal could be a potential target for neuroma prevention and/or therapeutic intervention. The other experiments demonstrated that infiltration of inflammatory cells and fibroblasts was observed within the traumatic neuromas. This evidence indicated that control of the BDNF-trkB pathway after nerve injury could affect both bone and nerve regeneration simultaneously.

**Fig 10:**
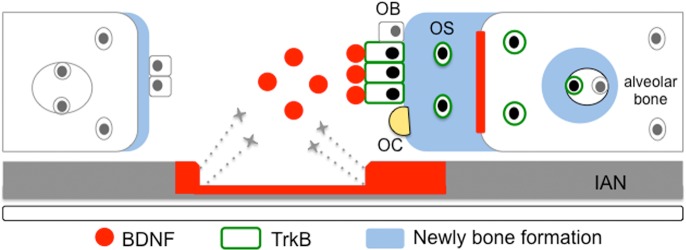
Graphic summary of the predicted mechanism of bone sclerosis after IAN. Peripheral nerve injury was caused by surgical maneuver, e.g., tooth extraction, and it invaded the inferior alveolar nerve, inducing local diffusion of BDNF at the lesion. BDNF stimulated the differentiation of osteoblasts through trkB receptor, resulting in sclerotic changes in the bone. As a result, osteogenesis and remodeling over the bone cavity wall were facilitated, making the wall thicker and resulting in isolation of the surrounding alveolar tissue. OB, osteoblast; OS, osteocyte; OC, osteoclast; IAN, inferior alveolar nerve.

In conclusion, we showed that BDNF stimulated the differentiation of osteoblast-lineage cells through trkB receptor. Exogenous BDNF also promoted osteosclerosis after cortical osteotomy in our *in vivo* experiment. Therefore, these results could explain the clinical findings of sclerotic changes in the alveolar bone near lesions due to peripheral nerve injury (Fig 10).

## Supporting Information

S1 Table The oligonucleotide primers used for RT-PCR. (TIF)
