# Supplementary figures and images for: Locally Produced BDNF Promotes Sclerotic Change in Alveolar Bone after Nerve Injury
Source: PLoS One. 2017 Jan 10;12(1):e0169201. doi: 10.1371/journal.pone.0169201 (PMC5224970; doi:10.1371/journal.pone.0169201)

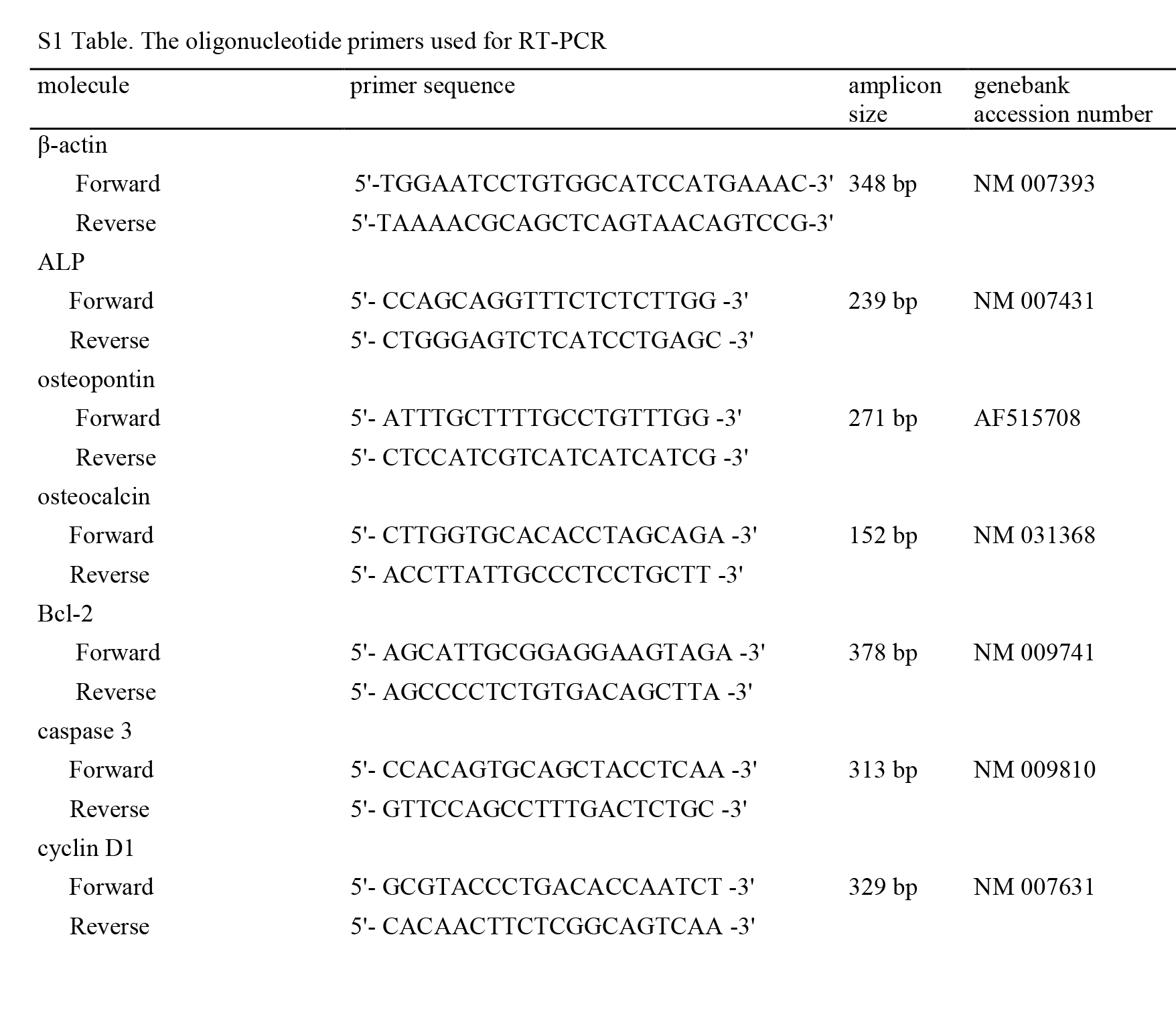

Supplement: S1 Table — (TIF) [file pone.0169201.s001.tif]
